# Aortic Pseudoaneurysm as an Unusual Cause of Vocal Palsy

**DOI:** 10.7759/cureus.44255

**Published:** 2023-08-28

**Authors:** Vetrivel G, Ankita Semwal, Sushil Raj Shrestha, Reshma Eugine, Amit Kumar Tyagi

**Affiliations:** 1 Otorhinolaryngology - Head & Neck Surgery, All India Institute of Medical Sciences, Rishikesh, IND

**Keywords:** left recurrent laryngeal nerve palsy, ortner’s syndrome, cardiovocal syndrome, vocal palsy, aortic pseudoaneurysm

## Abstract

The recurrent laryngeal nerve travels a variable course on either side due to the differences in the structures related during development. The nerve is at risk of injury due to a number of pathologies in any of these structures. We came across a very rare pathology causing vocal palsy in a 62-year-old male with hoarseness of voice. Laryngoscopy examination showed left vocal cord palsy without any obvious laryngeal mass. Contrast-enhanced computed tomography study of the neck and chest revealed aortic arch pseudoaneurysm with left vocal cord palsy.

## Introduction

Recurrent laryngeal nerve (RLN) is prone to injury at multiple anatomical sites due to its relation with a number of structures related to it starting from the cranial cavity, neck, and chest. Cardiovocal (Ortner’s) syndrome is related to intrinsic cardiac diseases [1]. To date, very few cases have been reported on pseudoaneurysm causing cardiovocal syndrome [2]. We are presenting the aortic pseudoaneurysm causing left RLN palsy that we came across in our institute.

## Case presentation

A 62-year-old male presented to the outpatient department of otorhinolaryngology - head & neck surgery with complaints of change in voice for the past three months, which was insidious in onset and non-progressive, with no diurnal variation. He also complained of dull aching pain in the upper back for the past 20 days, which was insidious in onset, gradual progressive, non-radiating, and not associated with respiratory difficulty. There were no aggravating or relieving factors. There was no history of difficulty or pain during swallowing, trauma, chest pain, fever, cough, breathlessness, night sweats, evening rise of temperature, or blood-stained sputum. He also does not have any comorbidities. The patient was a known tobacco smoker for many years. There was no history of any neck exploration or previous surgery. A thorough head and neck examination was done. Laryngeal endoscopy revealed a small cyst in the left vallecula and left true vocal cord palsy and no event of aspiration during the study (Video 1). The rest of the head and neck examination was unremarkable.

**Video 1:** Laryngeal endoscopy showing left vallecular cyst and left true vocal cord palsy due to aortic pseudoaneurysm.

**Figure 1:**
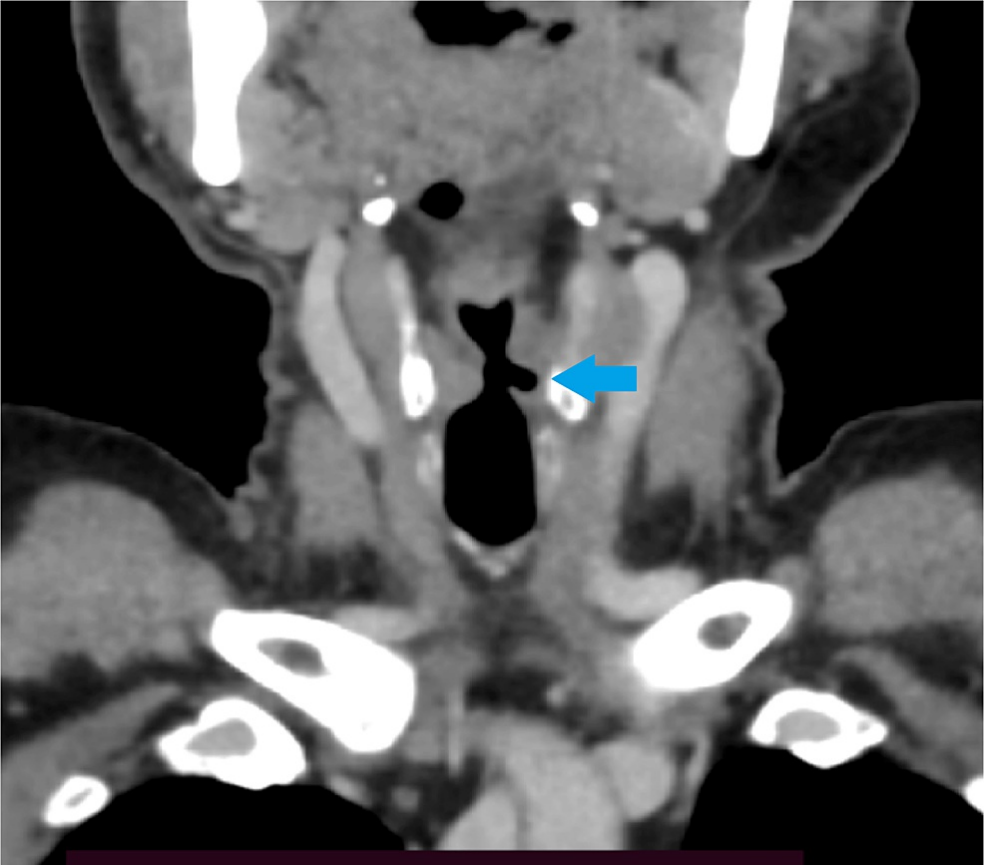
Contrast-enhanced computed tomography of the neck in the coronal section showing the dilated laryngeal ventricle, dilation of pyriform sinus, and lateralized true vocal fold on the left side consistent with the sail sign. Dilated left laryngeal ventricle (arrow).

**Figure 2:**
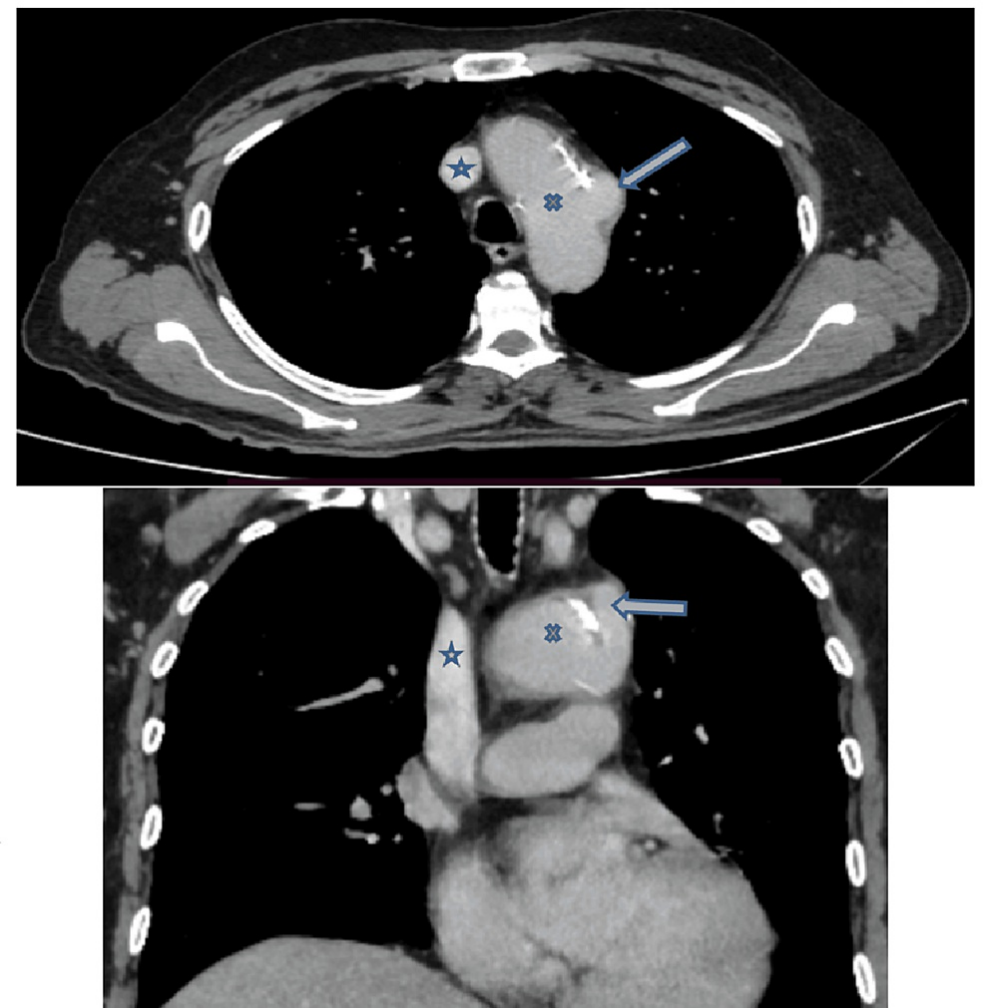
Computed tomography demonstrating the pseudoaneurysm of the aortic arch along with atherosclerotic changes. Pseudoaneurysm (arrow), aortic arch (cross), and superior vena cava (star).

As per the protocol, a contrast-enhanced computed tomography (CECT) from the base of the skull to the diaphragm was done in search of any pathology. It identified a non-enhancing, well-defined hypodense lesion, likely a vallecular cyst in the left vallecula. Imaging can very well demonstrate vocal cord paralysis showing dilatation of the ipsilateral pyriform sinus, laryngeal ventricle, and also the medialization of aryepiglottic fold and lateralization of the true vocal fold, referred to as sail sign (Figure 1) [3]. The imaging also revealed a focal contrast-filled outpouching arising from the arch of the aorta just distal to the origin of the left subclavian artery suggesting pseudoaneurysm. Computed tomography suggested left vocal cord palsy due to RLN compression by an aortic pseudoaneurysm. Atherosclerotic changes were noticed in the right innominate and left carotid arteries (Figure 2). The patient was referred to cardio-thoracic vascular surgery and planned for thoracic endovascular aortic repair. On follow-up, the patient denied undergoing the procedure, hence he was managed conservatively.

## Discussion

The course of RLNs is different on each side because of developmental factors. The nerve on the left side arises from the intrathoracic vagal nerve, traverses through the aortopulmonary window winding around the ligamentum arteriosum, and then coursing over a more straight course along the tracheoesophageal groove, usually passing behind the inferior thyroid artery and entering the larynx at the cricothyroid joint [1]. The nerve is more vulnerable to associated pathology because of more related structures. A literature search was performed with keywords, including pseudoaneurysm, vocal palsy, recurrent laryngeal nerve palsy, and cardiovocal syndrome, in PubMed, Google Scholar, Embase, Scopus, and Ovid. The search yielded only a few case reports, suggesting that pseudoaneurysm being the cause of left RLN palsy is infrequent. Cardiovocal (Ortner’s) syndrome is applied to RLN palsy related only to intrinsic cardiac diseases like valvular heart disease or cardiomegaly. This terminology does not apply to extrinsic lesions like aortic aneurysms and mediastinal tumors [1]. Cardiovascular disorders responsible for vocal cord palsy include aortic dissection, pseudoaneurysm, mitral stenosis, enlargement of the left atrium, congenital heart diseases, and pulmonary embolism [4,5]. To the best of our knowledge, very few articles report the incidence of RLN palsy due to the pseudoaneurysm of the aorta. The underlying mechanism is the loss of adventitia and periadventitial layers of the artery, thereby resulting in the protrusion of the medial and intimal layers [6]. Overall, cardiovocal syndrome accounts for 11% of RLN cases [1,7]. Compression of the left RLN in the vulnerable space, i.e., the aortopulmonary window beneath the aortic arch, can result from a pseudoaneurysm. In our case, there were no overt symptoms of an aneurysmal rupture. Very rarely, aortic aneurysms can cause vocal paralysis. Aortic aneurysms arise from multiple etiologies broadly classified into traumatic, degenerative, and infective causes. They can be treated with endovascular stent grafting techniques [8].

## Conclusions

Cardiovocal syndrome is characterized by vocal cord palsy brought on by different cardiovascular conditions. It is crucial to examine the patient's neck and chest if they have hoarseness in their voice since aortic pseudoaneurysm is a rare but significant cardiovascular cause of vocal cord palsy. The most crucial imaging technique to diagnose this condition and distinguish it from other cardiovascular conditions that can cause vocal cord palsy is the CECT of the neck and chest.
